# Monocyte and T Cell Immune Phenotypic Profiles Associated With Age Advancement Differ Between People With HIV, Lifestyle-Comparable Controls and Blood Donors

**DOI:** 10.3389/fimmu.2020.581616

**Published:** 2020-10-06

**Authors:** Davide De Francesco, Caroline A. Sabin, Peter Reiss, Neeltje A. Kootstra

**Affiliations:** ^1^Institute for Global Health, University College London, London, United Kingdom; ^2^Amsterdam institute for Global Health and Development, Amsterdam, Netherlands; ^3^Department of Global Health & Division of Infectious Disease, Amsterdam University Medical Centers, University of Amsterdam, Amsterdam, Netherlands; ^4^HIV Monitoring Foundation, Amsterdam, Netherlands; ^5^Department of Experimental Immunology, Amsterdam Infection and Immunity Institute, Amsterdam University Medical Centers, University of Amsterdam, Amsterdam, Netherlands

**Keywords:** HIV, aging, T cell, monocyte, immune activation, inflammation, immune dysfunction

## Abstract

**Motivation:**

People with HIV on successful antiretroviral therapy show signs of premature aging and are reported to have higher rates of age-associated comorbidities. HIV-associated immune dysfunction and inflammation have been suggested to contribute to this age advancement and increased risk of comorbidities.

**Method:**

Partial least squares regression (PLSR) was used to explore associations between biological age advancement and immunological changes in the T cell and monocyte compartment in people with HIV (n=40), comparable HIV-negative individuals (n=40) participating in the Comorbidity in Relation to AIDS (COBRA) cohort, and blood donors (n=35).

**Results:**

We observed that age advancement in all three groups combined was associated with a monocyte immune phenotypic profile related to inflammation and a T cell immune phenotypic associated with immune senescence and chronic antigen exposure. Interestingly, a unique monocyte and T cell immune phenotypic profile predictive for age advancement was found within each group. An inflammatory monocyte immune phenotypic profile associated with age advancement in HIV-negative individuals, while the monocyte profile in blood donors and people with HIV was more reflective of loss of function. The T cell immune phenotypic profile in blood donors was related to loss of T cell function, whereas the same set of markers were related to chronic antigen stimulation and immune senescence in HIV-negative individuals. In people with HIV, age advancement was related to changes in the CD4^+^ T cell compartment and more reflective of immune recovery after cART treatment.

**Impact:**

The identified monocyte and T cell immune phenotypic profiles that were associated with age advancement, were strongly related to inflammation, chronic antigen exposure and immune senescence. While the monocyte and T cell immune phenotypic profile within the HIV-negative individuals reflected those observed in the combined three groups, a distinct profile related to immune dysfunction, was observed within blood donors and people with HIV. These data suggest that varying exposures to lifestyle and infection-related factors may be associated with specific changes in the innate and adaptive immune system, that all contribute to age advancement.

## Introduction

The introduction of antiretroviral therapy (ART) for the treatment of human immunodeficiency virus type 1 (HIV-1) infection has dramatically reduced AIDS-associated morbidity and mortality, and greatly improved life expectancy of people with HIV (1–3). However, successfully treated people with HIV are now reported to have higher rates of age-associated comorbidities than the general population (1, 4–7), suggesting accentuated and possible even accelerated aging of people with HIV. The cause of the accentuated and/or accelerated aging in people with HIV remains to be fully elucidated, with factors like HIV associated chronic immune activation and dysfunction, ART toxicity and the higher prevalence of traditional risk factors each having been implicated to contribute to age-associated comorbidity development (8, 9).

In the general population aging is associated with immunological changes resulting in low grade inflammation and deterioration of the innate and adaptive immune system in the elderly (10–13). These age-related immunological changes resemble those seen during treated HIV-1 infection and include high levels of soluble inflammatory proteins, high levels of monocyte and T cell activation, T cell exhaustion and senescence, and low levels of naïve T cells (14–25). Moreover, HIV-1 associated changes in the immune system have also been associated with age-associated comorbidity development in people with HIV (26–28).

Previously, we demonstrated that both treated people with HIV and lifestyle-comparable HIV-negative individuals show signs of age advancement compared with blood donors (29). People with HIV exhibited incomplete immune recovery and increased immune activation/exhaustion compared to comparable HIV-negative controls (16). However, the levels of terminally differentiated T cells did not differ between people with HIV and comparable HIV-negative individuals, but were higher when compared to those of the age-matched blood donors (16). Interestingly, increased levels of cellular monocyte activation were observed in a subset of individuals, which was the case for both the people with HIV and the otherwise comparable HIV-negative individuals. Furthermore, this high level of monocyte activation was associated with greater inflammation, both systemically and in the CSF (17).

We now aim to extend these earlier findings by exploring associations between the previously observed biological age advancement on the one hand, and immunological changes in the T cell and monocyte compartments on the other hand in blood donors, people with HIV and comparable HIV-negative individuals participating in the Comorbidity in Relation to AIDS study (COBRA) (30).

## Materials and Methods

### Study Population

The COBRA study included 134 people with HIV on combination ART and 79 demographically and lifestyle comparable HIV-negative individuals from sites in the UK and the Netherlands with the aim to allow a detailed, prospective evaluation of the impact of HIV infection on the prevalence, incidence, and age at onset of age-associated comorbidities (30). Exclusion criteria were as follows: age under 45 years (50 years for participants recruited in the UK); self-reported intravenous drug use in the past 6 months; daily use of recreational drugs (with the exception of cannabis); excess alcohol intake (>48 units per week); (history of) confounding neurological diseases; severe head injury (loss of consciousness for >30 min); infections or tumors involving the central nervous system (CNS); current major depression (Patient Health Questionnaire-9 questionnaire score ≥15); and a contraindication to magnetic resonance imaging or lumbar puncture. All people with HIV were required to have had undetectable plasma HIV RNA (<50 copies/mL) for ≥12 months before enrolment.

For the present immunological sub-study, 40 people with HIV and 40 HIV-negative participants were randomly selected with equal numbers in each of the following age groups: 45–50 years, 51–55 years, 56–60 years, 61–65 years, 66–70 years, except for the oldest age category >70 years in which only a few individuals were available (16, 17). Materials from 35 blood donors were obtained from the Dutch national blood bank in Amsterdam, the Netherlands (https://www.sanquin.nl/en/) (16, 17). Blood donors were matched for age with the people with HIV and HIV-negative COBRA participants and were selected in such a way that the different COBRA age categories (other than the >70 years category) were equally represented. Blood donors had been screened for HIV, HBV, HCV, syphilis, and human T-lymphotropic virus (HTLV) infection, as a requirement for blood donation in the Netherlands. Moreover, potential blood donors are excluded from blood donation if they were determined to be at high-risk of blood-borne infections (based on a questionnaire regarding general and sexual health, medication use, sexual risk behavior, and travel (https://www.sanquin.nl/en/give-blood/).

### Biomarkers of Aging

A 10-item panel of biomarkers of aging identified by the MARK-AGE project (31, 32) were measured and described previously (29). Briefly, these biomarkers have been selected as best predictors of chronological age among nearly 400 candidate biomarkers in a population of approximately 3300 individuals aged between 30 and 74 years (mean age was 56 years) recruited from eight European countries.

Lycopene, and alpha-tocopherol in plasma were determined by high performance liquid chromatography with UV and fluorescence detection (33). Alpha-2-macroglobulin in plasma was measured using an autoanalyzer (DxC 800; Beckman-Coulter, Woerden, The Netherlands) by an immunoturbimetric method (Dialab, Wiener Neudorf, Austria) (34). Dehydroepiandrosterone sulfate, ferritin (women only), and prostate-specific antigen (men only) in plasma were analyzed using an immuno-analyzer (Access-2; Beckman–Coulter) (34). ELOVL2 and FHL2 DNA methylation in peripheral blood mononuclear cells (PBMCs) was analyzed using the Agena Bioscience’s EpiTYPER DNA methylation analysis technology (35). The N-glycans present on the proteins in plasma were released, labeled, and analyzed by DSA-FACE technology (36).

The biological age of each individual was derived separately for men and women as a linear combination of these biomarkers using the method and the weights described previously (29, 32). Age advancement for each individual was then defined as the difference between biological and chronological age (29). Positive age advancement is, therefore, indicative of more age-related changes than what would be expected in the average population, given the observed chronological age; negative age advancement would suggest less age-related changes.

### Immune Phenotyping

Cryopreserved peripheral blood mononuclear cells (PBMCs) from COBRA participants and blood donors were used for immune phenotyping as described previously (16, 17). PBMC were thawed and cell viability was analyzed by trypan blue staining and for FACS analysis cell viability was required to be >75%. PBMC were stained with conjugated monoclonal antibodies (mAbs) for 30 min at 4°C in the dark. Fluorescence was measured with the FACS Canto II (BD Biosciences). The proportion of cells and the mean fluorescence intensity (MFI) of the markers was determined using FlowJo 7.6 (TreeStar, Ashland, OR).

Monocyte subsets were defined based on the CD14 and CD16 expression: classical (CD14^+^CD16^−^), intermediate (CD14^+^CD16^+^), and non-classical (CD14^+^CD16^++^) monocytes. Within these subsets, the expression levels of activation markers (CD163, CD32, CD64, HLA-DR, CD38), T-cell costimulatory molecules (CD40 and CD86), and adhesion molecules (CD91, CD11c, and CX3CR1) were determined.

T cell subsets were determined by the expression of CD45RA, CD27 and CCR7 and were defined as naïve (CD45RA^+^CD27^+^CCR7^+^), central memory (CD45RA^−^CCR7^+^CD27^+^), transitional memory (CD45RA^−^CCR7^−^CD27^+^), effector memory (CD45RA^−^CCR7^−^CD27^−^), and terminally differentiated effector memory (TEMRA; CD45RA^+^CCR7^−^CD27^−^) within the total CD4^+^ or CD8^+^ T cell population. The proportion of naïve T cells expressing CD31 and CD127 (IL-7Rα) was determined. T cell activation and exhaustion was determined by the proportion of cells that were positive for both CD38 and HLA-DR or PD1 within the total CD4^+^ or CD8^+^ T cell population. Terminally differentiated T cells within the CD4^+^ or CD8^+^ population were defined as proportion of CD57 positive cells, proportion of cells negative for both CD27 and CD28, or the proportion of CD57 positive cells within the CD28−negative population.

The following directly conjugated mAbs were used for cell surface marker staining: CD14 PE-Cy7, CD16 eFluor 450, CD32 PerCP-eFluor 710, CD11c APC, CD27 APCeFluor780, CD4 APC eFluor780, CD127 APC eFluor780, and PD-1 PE (eBioscience, San Diego, CA); CD163 AlexaFluor 488 and CD86 PerCP (R&D Systems, Minneapolis, MN); CX3CR1 PerCP-Cy5.5 (BioLegend, San Diego, CA); CD3 V500, CD4 PE-Cy7, HLA-DR Fitc, CD38 PE, CD28 PerCP Cy5.5, CD45RA PE-Cy7, CD8 Pacific Blue, CD57 APC, CCR7 PE, CD27 PerCP Cy5.5, and HLA-DR V500 (BD Biosiences, San Jose, CA); CD38 PE and CD91 PE (BD); and CD31 APC, CD40 APC-H7, CD64 APC-H7, and CD8 Pacific Blue (BD Pharmingen, San Diego, CA).

### Statistical Analysis

Participant characteristics were summarized using median (interquartile range: IQR) or frequency count (percentage) and differences between people with HIV and both COBRA HIV-negative individuals and blood donors were assessed using χ^2^, Fisher’s exact and Wilcoxon rank‐sum tests, as appropriate. Age advancement within each group was reported as mean and 95% confidence intervals (CI); pairwise differences between groups were evaluated using the independent samples t-test.

Partial least squares regression (PLSR) was used to explore associations of monocyte and T cell markers, separately, with age advancement. PLSR identifies the linear combination of all biomarkers within the set that best predicts (correlates with) age advancement (37). Variable Importance in Projection (VIP) values for each biomarker were calculated with the sign depending on the direction of the association (38). VIP values rank biomarkers on the basis of their importance in making up the linear combination that best predicts age advancement; these were used to identify biomarkers within each set (monocyte and T cell population) with the strongest association with age advancement.

For each set of biomarkers, a first PLSR was run in the three groups (people with HIV, HIV-negative and blood donors) combined. The correlation between the score obtained by PLSR (i.e. the combination of all biomarkers within the set with weights estimated by PLSR) and age advancement was evaluated using the Spearman’s correlation coefficient. Subsequently, we ran a separate PLSR within each of the three study groups. All statistical analyses were performed using R v3.6.0.

## Results

### Baseline Characteristics of COBRA Participants and Blood Donors

Baseline characteristics of the 40 people with HIV, 40 COBRA HIV-negative individuals and 35 blood donors have been described previously (16, 17) and are summarized in **Table 1**. Briefly, people with HIV and COBRA HIV-negative individuals had a median (IQR) age of 59 (54–64) years, blood donors had a median (IQR) age of 59 (52–65) years. People with HIV had been diagnosed with HIV for a median (IQR) of 13.9 (9.1–18.6) years, had been on ART for a median (IQR) of 12.2 (7.9–16.9) years and had spent a median (IQR) of 8.0 (5.3–10.9) years with an undetectable plasma viral load. Despite long-term suppression of HIV-replication by combination ART, people with HIV exhibited lower CD4^+^ and higher CD8^+^ T-cell counts compared to COBRA HIV-negative individuals (both p’s<0.001).

**Table 1:** Characteristics of study participants.

Variable, n (%) or median (IQR)	COBRA participants^4^		p^1^	Blood donors (n = 35)	p^2^	p^3^
	People with HIV (n = 40)	HIV-negative (n = 40)				
Age [years]	59 (54, 64)	59 (54, 64)	0.96	59 (52, 65)	0.83	0.80
Gender						
Female	4 (10.0%)	3 (7.5%)	0.69	17 (48.6%)	<0.001	<0.001
Male	36 (90.0%)	37 (92.5%)		18 (51.4%)		
Ethnicity						
Black-African	5 (12.5%)	1 (2.5%)	0.20	n/a		
White	35 (87.5%)	39 (97.5%)		n/a		
Sexuality						
MSM	27 (67.5%)	28 (70.0%)	0.75	n/a		
Bisexual	5 (12.5%)	3 (7.5%)		n/a		
Heterosexual	8 (20.0%)	9 (22.5%)		n/a		
BMI [kg/m^2^]	24.8 (22.5, 27.2)	26.2 (23.7, 31.1)	0.07	n/a		
Years of education	14 (12, 16)	16 (14, 18)	0.02	n/a		
Smoking status						
Current smoker	12 (30.0%)	10 (25.0%)	0.88	n/a		
Ex-smoker	18 (45.0%)	19 (47.5%)		n/a		
Never smoked	10 (25.0%)	11 (27.5%)		n/a		
Alcohol consumption						
Current consumption	28 (70.0%)	34 (87.2%)	0.18	n/a		
Previous consumption only	7 (17.5%)	3 (7.7%)		n/a		
Never consumed alcohol	5 (12.5%)	2 (5.1%)		n/a		
Current recreational drug use	10 (25.0%)	9 (22.5%)	0.79	n/a		
Current/past ID use	1 (2.5%)	0 (0.0%)	0.31	n/a		
Chronic HBV infection	3 (7.7%)	0 (0.0%)	0.12	0 (0.0%)	0.24	n/a
Chronic HCV infection	1 (2.6%)	0 (0.0%)	0.31	0 (0.0%)	0.34	n/a
CMV infection	38 (95.0%)	31 (77.5%)	0.02	8 (22.9%)	<0.001	<0.001
Total anti-CMV IgG [AU]	50.9 (23.5, 108.6)	23.9 (13.8, 87.8)	0.03	11.3 (10.2, 16.8)	0.003	0.09
High avidity anti-CMV IgG [AU]	30.7 (13.0, 57.0)	13.3 (8.2, 39.7)	0.05	10.7 (10.0, 13.2)	0.05	0.42
CD4^+^ T cell count [cells/µL]	589 (470, 800)	961 (759, 1233)	<0.001	n/a		
CD8^+^ T cell count [cells/µL]	762 (636, 1029)	488 (364, 621)	<0.001	n/a		
CD4^+^:CD8^+^ T cell count ratio	0.80 (0.61, 1.13)	1.95 (1.33, 2.83)	<0.001	3.1 (2.3, 4.2)	<0.001	<0.001
Years since HIV diagnosis	13.9 (9.1, 18.6)	n/a		n/a		
Nadir CD4^+^ T cell count [cells/µL]	180 (60, 230)	n/a		n/a		
Duration of cART therapy [years]	12.2 (7.9, 16.9)	n/a		n/a		
Years with plasma HIV RNA <200 copies/mL	8.0 (5.3, 10.9)	n/a		n/a		
Prior AIDS	15 (37.5%)	n/a		n/a		

p^1^, People with HIV vs. COBRA HIV-negative participants; p^2^, people with HIV vs. blood donors; p^3^, COBRA HIV-negative participants vs. blood donors; ^4^median (IQR) or frequency (%) of the variable are reported for continuous and categorical variables, respectively; ID, injection drug; information regarding, alcohol consumption, hepatitis B status, CD4^+^, CD8^+^ and CD4^+^:CD8^+^ T cell count was not available for 1 COBRA participant.

### Age Advancement

**Figure 1:**
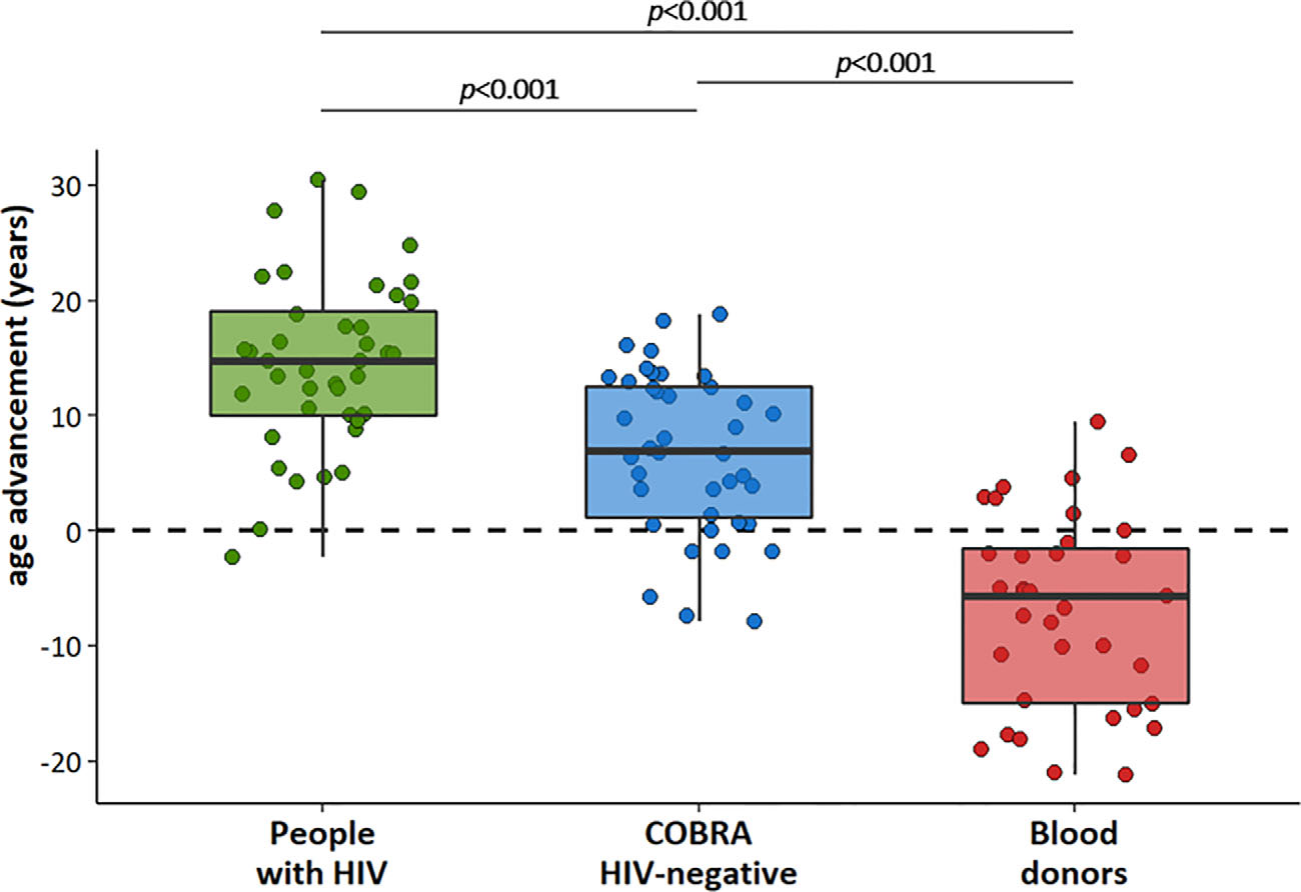
Age advancement in people with HIV (n=40), COBRA HIV-negative individuals (n=40) and blood donors (n=35).

Similar to our previous observations (29), biological age as determined using the MARK-AGE algorithm (32) in the subset of individuals selected for this immunological substudy was significantly greater than chronological age by a mean (95% CI) of 14.6 (12.2–16.9) years in people with HIV and by 6.9 (4.6–9.1) years in COBRA HIV-negative participants, whereas in blood donors biological age was lower than chronological age by a mean of 7.0 (4.1–9.9) years (**Figure 1**). All pairwise differences between the three groups were significant (all p’s <0.001).

### Associations Between the Monocyte Immune Phenotypic Profile and Age Advancement

Monocytes from participants of the three study groups were analyzed for the percentages of classical, intermediate and non-classical monocyte subsets and the expression levels of activation markers (CD163, CD32, CD64, HLA-DR, CD38), T-cell costimulatory molecules (CD40 and CD86), and adhesion molecules (CD91, CD11c, and CX3CR1) (17).

**Figure 2:**
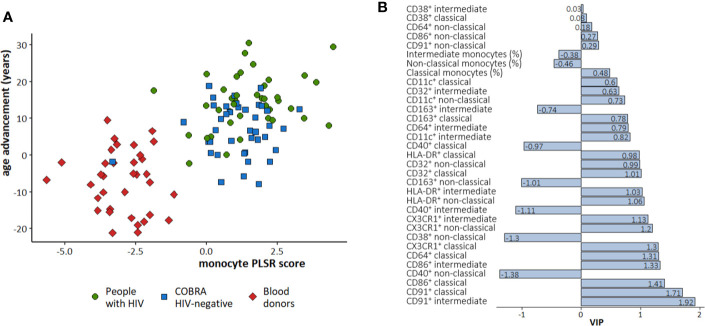
Partial least squares regression (PLSR) to predict age advancement with monocyte markers in the three study groups combined. **(A)** Scatter plot of the PLSR score obtained and age advancement. Green circle: People with HIV; Bleu square: COBRA HIV-negative individuals; Red diamond: Blood donors. **(B)** Variable Importance in Projection (VIP) values for each monocyte marker. A negative VIP indicates that decreased levels of the specific marker is associated with increased age advancement. A positive VIP indicates that increased levels of the specific markers is associated with increased age advancement.

The monocyte PLSR score identified in the three groups combined was strongly associated with age advancement [r (95% CI): 0.67 (0.55–0.76)] and discriminated between COBRA participants and blood donors (**Figure 2A**). A higher expression of CX3CR1 and CD64 on classical monocytes, and CD86 and CD91 on classical and intermediate monocytes, and a lower expression of CD38 and CD40 on non-classical monocytes appeared to best predict age advancement (**Figure 2B**).

### The Monocyte Immune Phenotypic Profile Associated With Age Advancement Differs Between People With HIV, Comparable HIV-Negative Individuals and Blood Donors

**Figure 3:**
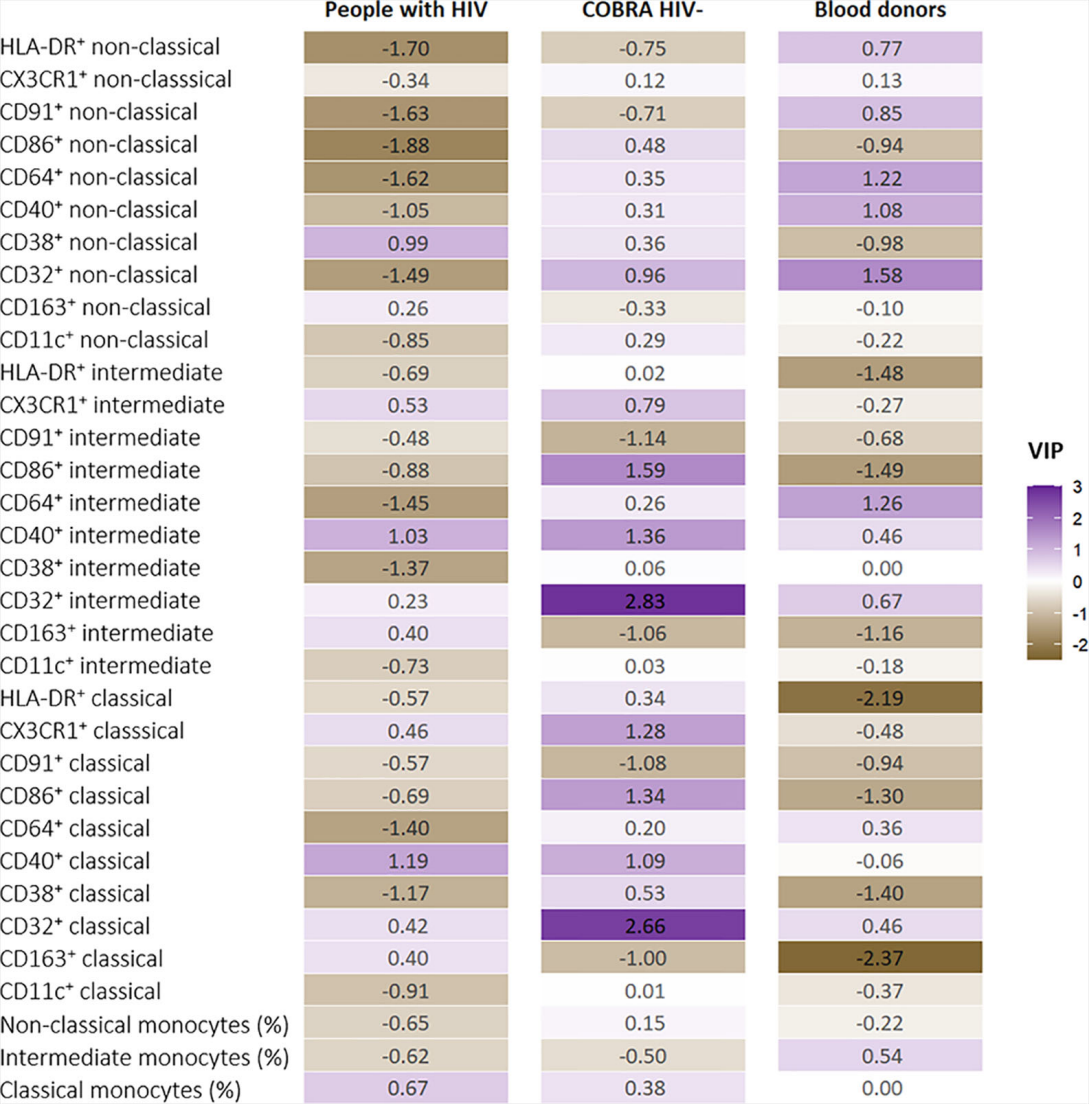
Variable Importance in Projection (VIP) values for the monocyte markers in the partial least squares regression (PLSR) run separately in people with HIV (n=40), COBRA HIV-negative individuals (n=40) and blood donors (n=35). VIP scores are color-coded ranging from brown for a negative VIP to purple for a positive VIP.

**Figure 3** displays the VIP for each monocyte marker in the analyses stratified by study group, and shows that age advancement was associated with different sets of monocyte markers within each group. Among people with HIV, markers most strongly associated with age advancement were the expression of HLA-DR, CD91, CD86, CD32 on non-classical monocytes, CD38 on intermediate monocytes and CD64 on all monocytes, with lower expressions being associated with greater age advancement. In COBRA HIV-negative individuals, increased expression of CD86, CD32, and CD40 on intermediate monocytes and CD86, CD32 and CX3CR1 on classical monocytes showed the strongest associations with age advancement. Finally, among blood donors, increased expression of CD32 on non-classical monocytes and CD64 on intermediate and non-classical monocytes, and decreased expression of HLA-DR, CD86 and CD163 on classical and intermediate monocytes and CD38 on classical monocytes appeared to be associated with greater age advancement.

### Associations Between the T Cell Immune Phenotypic Profile and Age Advancement

CD4^+^ or CD8^+^ T cell populations of participants of the three study groups were analyzed for the proportion of naïve (CD45RA^+^CD27^+^CCR7^+^), central memory (CD45RA^−^CCR7^+^CD27^+^), transitional memory (CD45RA^−^CCR7^−^CD27^+^), effector memory (CD45RA^−^CCR7^−^CD27^−^), terminally differentiated effector memory (TEMRA; CD45RA^+^CCR7^−^CD27^−^), naïve cells expressing CD31 and CD127 (IL-7Rα), activation and exhaustion (HLA-DR^+^/CD38^+^ and PD1^+^), terminally differentiation (CD57^+^, CD27^-^/CD28^-^, and CD57^+^ of CD28^−^ population) and regulatory T cells (16).

**Figure 4:**
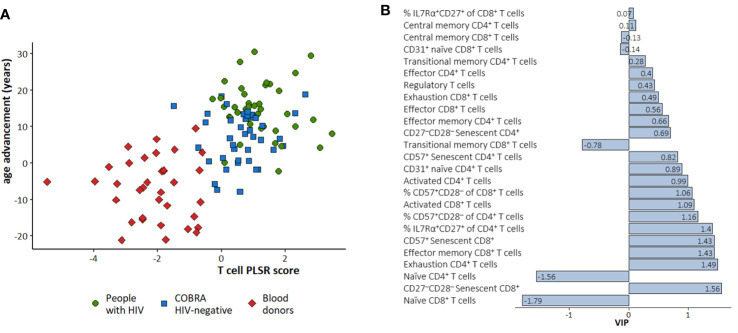
Partial least squares regression (PLSR) to predict age advancement with T cell markers in the three study groups combined. **(A)** Scatter plot of the PLSR score obtained and age advancement. Green circle: People with HIV; Bleu square: COBRA HIV-negative individuals; Red diamond: Blood donors. **(B)** VIP values for each T cell marker. A negative VIP indicates that decreased levels of the specific marker is associated with increased age advancement. A positive Variable Importance in Projection (VIP) indicates that increased levels of the specific markers is associated with increased age advancement.

The PLSR score obtained from T cell phenotypic and activation markers in the three groups combined correlated well with age advancement [r (95% CI) = 0.62 (0.49, 0.72)], and separated blood donors from the COBRA participants (**Figure 4A**). The strongest predictors for age advancement were lower percentage of naïve CD4^+^ and CD8^+^ cells, increased proportion of IL-7Ra expressing CD4^+^ naïve cells, exhausted CD4^+^ cells, effector memory CD8^+^ cells and senescent (CD57^+^ and CD27^-^CD28^-^) CD8^+^ cells (**Figure 4B**).

### The T Cell Immune Phenotypic Profile Associated With Age Advancement Differs Between People With HIV, Comparable HIV-Negative Individuals and Blood Donors

**Figure 5:**
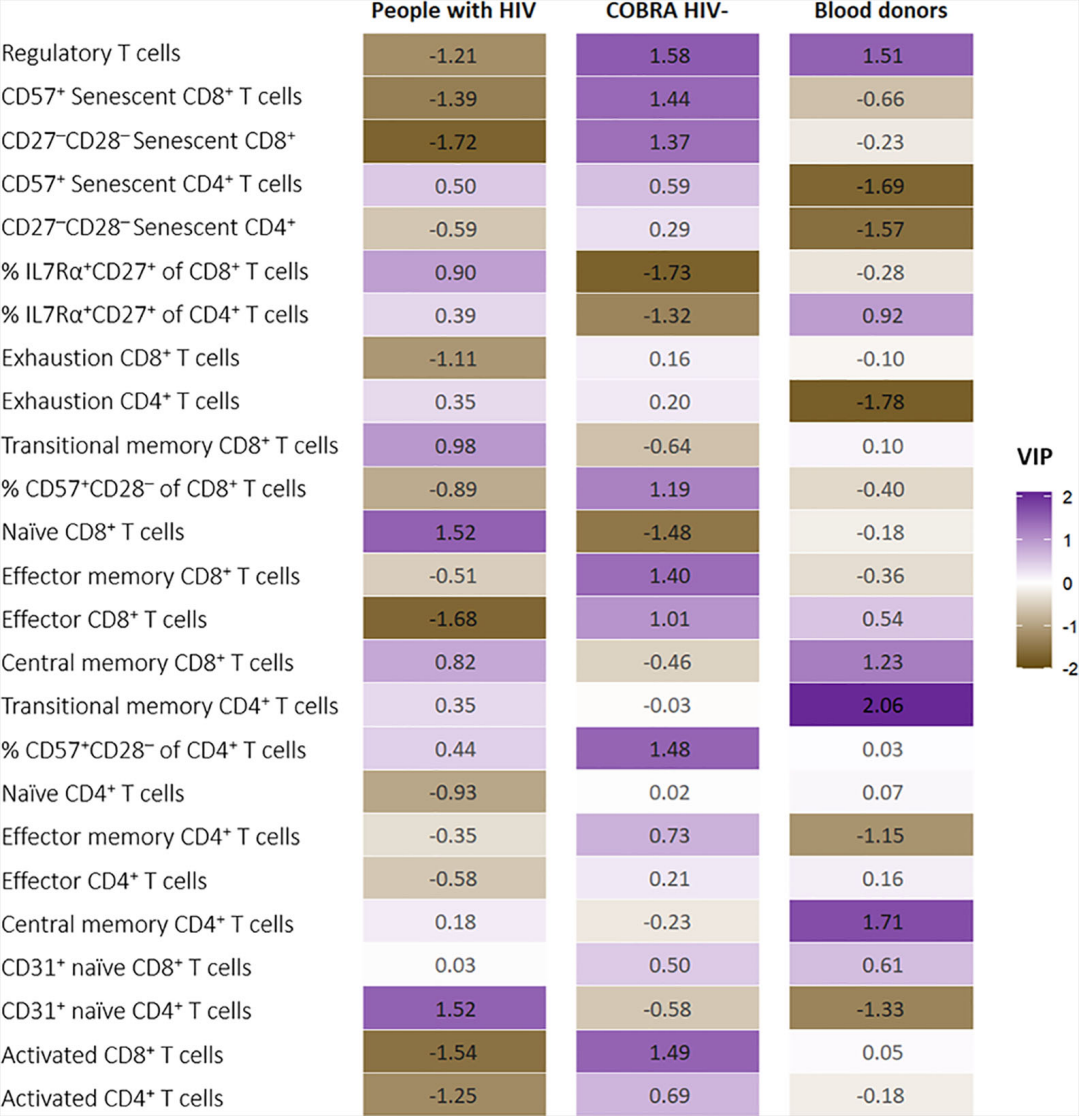
Variable Importance in Projection (VIP) values for the T cell markers in the partial least squares regression (PLSR) run separately in people with HIV (n=40), COBRA HIV-negative individuals (n=40) and blood donors (n=35). VIP scores are color-coded ranging from brown for a negative VIP to purple for a positive VIP.

T cell phenotypic and activation markers associated with biological age advancement differed significantly in the PLSR analysis stratified by group (**Figure 5**): Among people with HIV, a higher proportion of T regulatory and memory cells (central memory CD4^+^ and CD8^+^, transitional memory CD8^+^) was associated with age advancement, whereas decreased proportions of CD31 expressing naïve CD4^+^ cells, exhausted and senescent (CD57^+^ and CD27^-^CD28^-^) CD4^+^ cells were associated with age advancement. Among COBRA HIV-negative individuals, greater proportions of T regulatory, activated CD8^+^, effector memory CD8^+^, senescent (CD57^+^ and CD27^-^CD28^-^) CD8^+^ and CD57^+^ of CD28^-^ CD4^+^ cells, and lower proportions of naïve CD8^+^ and IL-7Ra expressing CD4^+^ and CD8^+^ naïve cells were associated with greater age advancement. Finally, greater proportions of naïve CD8^+^ and CD31 expressing naïve CD4^+^ cells, and lower proportions of T regulatory, activated CD4^+^ and CD8^+^, CD8^+^ effector cells and senescent (CD57^+^ and CD27^-^CD28^-^) CD8^+^ cells were associated with greater age advancement among blood donors.

## Discussion

Age advancement was associated with a monocyte immune phenotypic profile of which higher expression of CX3CR1, CD64, CD86, and CD91, and a lower expression of CD38 and CD40 on different monocyte subsets were the best predictors of age advancement in our combined study population consisting of people with HIV, HIV-negative and blood donors. Monocytes are both important regulators and effectors in inflammation, and an increased proportion of inflammatory or nonclassical (CD14^+^CD16^++^) monocytes producing high levels of cytokines has been associated with aging in the general population (39, 40). Moreover, monocyte functions like phagocytosis, antigen presentation and TLR signaling, decrease with age (39, 41–43). The identified monocyte profile strongly reflects differences in the expression of monocyte markers between the groups as we previously reported (17). Although, no functional analysis has been performed, the monocyte markers have previously been linked to inflammation and control of inflammation. CX3CR1 is the chemoattractant cytokine CX3CL1 receptor, and has been shown to play an important role in the modulation of inflammatory responses (44) and its expression is increased in HIV infection (45, 46). CD64 is the high-affinity IgG receptor FcγR, upregulated during inflammation (47, 48). CD86 provides costimulatory signals necessary for T-cell activation and survival, and has an anti-inflammatory role *in vivo* (49). CD91 is the low-density lipoprotein receptor-related protein 1, and is a major regulator of inflammation (50–52). CD91 does not change with age, but has been shown to be upregulated during HIV-1 infection to control inflammation and maintain overall immune function. The increased expression of these molecules on monocyte subsets of COBRA participants may be a response to overall higher ongoing inflammation.

CD38 expression on monocytes can be induced by pro-inflammatory cytokines (53). During viral infection down regulation of CD38 has been observed, possibly to reduce virus‐induced hyperinflammation (54). CD40, a member of the tumor necrosis factor (TNF) receptor family of cell surface proteins, provides strong costimulatory signal to prime T cells. On monocytes, CD40 stimulation induces the production of inflammatory cytokines and chemokines, and matrix metalloproteinases and induces more potent antigen presentation (55). Low CD40 expression has been observed in HIV infection (25). Therefore, CD38 and CD40 expression on monocytes of COBRA participants may be down regulated in response to viral infections and a high inflammatory environment. Changes in the monocyte profile were not associated with CMV infection, however it cannot be excluded that CMV infection contributes to the observed monocyte profile.

In agreement with earlier studies in the general population (11, 15, 56, 57), increased proportions of terminal differentiated and senescent of CD8^+^ T cells were strongly related to age advancement in our overall study population. These terminally differentiated CD8^+^ T cells have lost the expression of costimulatory molecules like CD27 and CD28, and are defective in T cell receptor signaling and proliferative capacity. However these cells do release cytokines and thereby contribute to age associated inflammation (11, 15, 56–58). Previously we reported that CMV infection was more prevalent in COBRA participants as compared to blood donors and that terminal differentiation and senescence in the CD8^+^ T cell compartment was strongly associated with CMV infection (16, 18). The identified T cell immune phenotypic profile was indeed associated with CMV infection (r 2.1; 95%CI 1.5-2.6; p<0.001), confirming that CMV infection is a strong driver of age related immune senescence and terminal differentiation of CD8^+^ T cells (11, 15, 56, 57). This also indicates that the CMV status may be a strong predictor of age advancement.

Within the CD4^+^ T cell compartment, increasing numbers of regulatory T cells are associated with aging, resulting in the suppression of immune responses in the elderly (59, 60). In contrast, in our study population the proportion of regulatory T cells was not a strong predictor of age advancement. We observed that increased PD1 expression, a marker of T cell exhaustion and activation (61) in the CD4^+^ T cell population was associated with age advancement. This may be indicative of a higher lifetime infection burden and antigen exposure in COBRA participants both with and without HIV as compared to blood donors (62). Similarly, this may also explain the association between age advancement and the increased proportion of CD4^+^ naïve T cells expressing the IL-7 receptor, which is indicative of homeostatic proliferation to maintain the CD4^+^ T cell pool especially during HIV infection (63). Moreover, increased proportions of CD4^+^ and CD8^+^ naïve T cells seem to be negatively associated with age advancement, probably reflecting maintenance of this population in the blood donors.

Both the monocyte and the T cell profile related to age advancement was able to separate blood donors from COBRA participants which is in line with the generally negative age advancement observed for this group, as opposed to the generally positive age advancement seen in both COBRA participants with and without HIV. By virtue of the study’s design (30), COBRA HIV-positive and HIV-negative participants were comparable with regard to many lifestyle-related factors, and thereby generally at higher risk for blood borne infections than blood donors (62). Indeed, blood donors in the Netherlands are specifically selected for their low risk of blood borne infections regarding their general and sexual health based on medication use, sexual risk behavior, and global travel. We also observed a lower CMV prevalence (22.9%) in blood donors as compared to the general population (64), which may explain the negative age advancement of this group using the MARK-AGE algorithm which is based on the general population [described in (32)].

Of note, within each of our three study groups, we identified a different monocyte and T cell immune phenotypic profile to be associated with age advancement.

Overall, blood donors showed no age advancement and even lower biological than chronological age, but when present age advancement within this group was related to decreased expression of HLA-DR and the scavenger receptor CD163 on classical monocytes which may be reflective of a decrease in monocyte function. In contrast to earlier reports (39, 40), no association between age advancement and increased proportion of inflammatory or nonclassical (CD14^+^CD16^++^) monocytes was observed in our blood donors. The association between age advancement and increased expression of HLA-DR, CD91, CD64, CD40 and CD32 on nonclassical monocytes in this group may nonetheless be reflective of a more inflammatory phenotypic of these cells.

COBRA participants generally showed a significantly higher age advancement as compared to blood donors, and additionally the monocyte immune phenotypic profile associated with age advancement seemed to be strongly affected by HIV. In COBRA HIV-negative participants, the increased expression of the low-affinity IgG receptor FcγR CD32 on intermediate and classical monocytes may be indicative of age associated monocyte activation and inflammation, while the overall decreased expression of markers like HLA-DR, CD91, CD86, CD32 on non-classical monocytes in people with HIV may be more reflective of a loss of monocyte functions such as antigen presentation, costimulation, phagocytosis and control of inflammation (39, 42, 49–52). HIV infection has a strong effect on the age associated monocyte immune phenotypic profile, resulting in the down regulation of several membrane markers affecting monocyte functionality.

The monocyte immune phenotypic profile associated with age advancement differed between the two HIV-negative groups. While the overall increased expression of CD32, CD40, CD86 and CX3CR1 on classical and intermediate monocytes of HIV-negative COBRA may be indicative of age-associated monocyte activation and inflammation, the decreased expression of CD163, CD86 and HLA-DR on these monocyte populations of blood donors are more reflective of age-associated loss of monocyte function. These data suggest that group specific lifestyle-related factors like smoking and recreational drug use, sexual behavior, and prevalence of infections (syphilis, CMV, HBV, HHV-8, human herpesvirus type 8 (HHV-8), herpes simplex virus (HSV)) strongly contribute to the monocyte immune phenotypic profile associated with the advancement of biological aging.

In the general population aging in the T cell compartment is characterized by accumulation of terminally differentiated and senescent CD8^+^ T cells and increasing numbers of regulatory T cells (11, 15, 56, 57, 59, 60).

In our blood donors age advancement was however not associated with the accumulation of senescent and terminally differentiated (effector and effector memory) CD8^+^ T cells, which could at least in part be explained by the low seroprevalence of CMV infection in this group as compared to the general Dutch population (64). The age associated increased proportion of CD8^+^ naïve cells, IL-7R expression within the naïve T cell and CD31 expression within naïve CD4^+^ T cells in blood donors indicate that increased homeostatic proliferation and egress of naïve cells from the thymus are required to maintain the peripheral naïve T cell pool (63, 65). Moreover, the age associated decreased proportion of regulatory T cells, activated CD4^+^ and CD8^+^ T cells in blood donors are suggestive of only low level immune activation and antigen exposure. This indicates that the T cell immune phenotypic profile associated with age advancement in blood donors is likely reflective of loss of T cells and T cell function during aging. Interestingly, a similar T cell profile was inversely associated with age advancement in COBRA HIV-negative participants, and is likely a manifestation of high CMV prevalence and immune activation due to lifestyle related factors and higher risk for blood borne infections than blood donors (62).

In people with HIV, age advancement was mainly correlated to changes in the CD4^+^ T cell population, the major target cell for HIV infection. Increased age advancement was associated with decreased proportions of senescent, exhausted, and effector memory CD4^+^ T cells, and increased proportions of central memory and transitional CD4^+^ T memory cells. This suggests that aging of people with HIV is associated with an accelerated loss of differentiated and exhausted CD4 cells that can quickly respond to antigen, produce effector cytokines and have a high turn-over rate (66, 67). Moreover, the association between age advancement and an increased proportion of regulatory T cells in people with HIV is indicative of suppression of immune activation, similar to observations in the elderly (59, 60) Therefore, the correlation between changes within the CD4^+^ T cell population and age advancement in people with HIV are likely representing higher CD4^+^ T cell turn-over and elevated levels of immune activation.

Monocyte and T cell immune phenotypic analysis as described here is laborious and therefore the sample size was limited to 35 blood donors and 40 participants for each of the COBRA study groups. This restricted our ability to detect small differences between the groups as well as within each group. This also limited our ability to differentiate the independent effects of viral co-infections (CMV, HCV, HBV), lifestyle-related factors (e.g. smoking, substance use), and exposure to antiretroviral drug regimens on the immune phenotypic profile in relation to age advancement. Moreover, we cannot rule out the possibility of other unmeasured differences between the groups which may impact on the immune phenotypic profiles observed. Another limitation is the cross-sectional nature of our study, and longitudinal studies will be necessary to evaluate whether the identified immune phenotypic profiles are persistent and may be predictive of accelerated or accentuated aging. Due to the cross cross-sectional nature of the study and the lack of functional assays, this study is mainly descriptive.

In conclusion, we identified an immunological profile in monocytes and T cells that is associated with age advancement as determined by the MARK-AGE algorithm, in the combined study population of people with HIV, HIV-negative people with similar lifestyles and blood donors. The identified monocyte and T cell immune phenotypic profile was strongly related to inflammation and chronic antigen exposure respectively, confirming a role of both the innate and adaptive immune system in the aging process. The identified immune phenotypic profile in monocytes and T cells was mainly based on differences between blood donors and COBRA participants as previously reported (16, 17) and likely related to differences in lifestyle related factors.

However, a different monocyte and T cell immune phenotypic profile was related to age advancement within each of the three groups, with an inflammatory monocyte immune phenotypic profile being associated with age advancement in HIV-negative COBRA participants, but a monocyte profile more reflective of loss of function being seen in blood donors and people with HIV. The identified T cell immune phenotypic profile associated with age advancement had opposite effects in blood donors and HIV-negative COBRA participants. While the T cell immune phenotypic profile in blood donors was related to loss of T cell function, the same set of markers were related to immune senescence and chronic antigen stimulation in HIV-negative COBRA participants. In people with HIV age advancement was mainly correlated with changes in the CD4^+^ T cell population and reflective of higher levels of immune activation and CD4^+^ T cell turn-over. We observed that biological aging was associated with changes in the innate and adaptive immune system related to inflammation, immune activation and dysfunction. The observed immunological changes were group specific indicating that the profile may change depending on differences in life-style, HIV infection, and exposures to other (viral) infections and underlying chronic conditions across the lifespan.

## Data Availability Statement

The datasets presented in this article are not readily available. Data sharing has been restricted by the Medisch Ethische Toetsingscommissie and the UK National Research Ethics Service (NRES) because the data underlying this study contains very sensitive and potentially identifying information. Requests for data sharing can be made on a case-by-case basis following submission of a concept sheet as per instructions on the project website (http://fp7-cobra.eu/). Once submitted the proposed research/analysis will undergo review by the COBRA Steering Committee for evaluation of the scientific value, relevance to the study, design and feasibility, statistical power and overlap with existing projects. If the proposed analysis is for verification/replication, data will then be made available. If the proposed research is for novel science, upon completion of the review, feedback will be provided to the proposer(s). In some circumstances, a revision of the concept may be requested. If the concept is approved for implementation, a writing group will be established consisting of the proposers (up to 3 persons that were centrally involved in the development of the concept) and members of the COBRA group (or other appointed cohort representatives). All persons involved in the process of reviewing these research concepts are bound by confidentiality. Medisch Ethische Toetsingscommissie, Academisch Medisch Centrum, Universiteit van Amsterdam Academisch Medisch Centrum, XT4-140 Meibergdreef 9, 1105 AZ Amsterdam, The Netherlands. UK National Research Ethics Service (NRES), Charing Cross Hospital, Fulham W6 8RF, London, UK.

## Ethics Statement

The studies involving human participants were reviewed and approved by Institutional review board of the Amsterdam University Medical Centers (location AMC; reference number NL 30802.018.09), the UK Research Ethics Committee (Stanmore, England, reference number 13/LO/0584), and the Ethics Advisory Body of the Sanquin Blood Supply Foundation in Amsterdam. The patients/participants provided their written informed consent to participate in this study.

## Author Contributions

DF contributed to study concept and design, literature search, data analysis and interpretation, figures, and writing of the manuscript. CS contributed to study concept and design, data collection and interpretation, and critical revision of manuscript. PR contributed to study concept and design, provided data, data collection and interpretation, and critical revision of manuscript. NK contributed to study concept and design, literature search, provided data, data interpretation, and writing of the manuscript. All authors contributed to the article and approved the submitted version.

## Funding

The COBRA project has received funding from the European Union’s Seventh Framework Programme for research, technological development and demonstration under grant agreement no 305522. The funders had no role in study design, data collection and analysis, decision to publish, or preparation of the manuscript.

## Conflict of Interest

The authors declare that the research was conducted in the absence of any commercial or financial relationships that could be construed as a potential conflict of interest.

## References

[1] Guaraldi G Orlando G Zona S Menozzi M Carli F Garlassi E Premature age-related comorbidities among HIV-infected persons compared with the general population. Clin Infect Dis (2011) 53(11):1120–6. 10.1093/cid/cir627 21998278

[2] Wada N Jacobson LP Cohen M French A Phair J Munoz A Cause-specific life expectancies after 35 years of age for human immunodeficiency syndrome-infected and human immunodeficiency syndrome-negative individuals followed simultaneously in long-term cohort studies, 1984-2008. Am J Epidemiol (2013) 177(2):116–25. 10.1093/aje/kws321 PMC3590031 23287403

[3] Weber R Ruppik M Rickenbach M Spoerri A Furrer H Battegay M Decreasing mortality and changing patterns of causes of death in the Swiss HIV Cohort Study. HIV Med (2013) 14(4):195–207. 10.1111/j.1468-1293.2012.01051.x 22998068

[4] Effros RB Fletcher CV Gebo K Halter JB Hazzard WR Horne FM Aging and infectious diseases: workshop on HIV infection and aging: what is known and future research directions. Clin Infect Dis (2008) 47(4):542–53. 10.1086/590150 PMC3130308 18627268

[5] Hasse B Ledergerber B Furrer H Battegay M Hirschel B Cavassini M Morbidity and aging in HIV-infected persons: the Swiss HIV cohort study. Clin Infect Dis (2011) 53(11):1130–9. 10.1093/cid/cir626 21998280

[6] Pathai S Bajillan H Landay AL High KP Is HIV a model of accelerated or accentuated aging? J Gerontol A Biol Sci Med Sci (2014) 69(7):833–42. 10.1093/gerona/glt168 PMC4067117 24158766

[7] Schouten J Wit FW Stolte IG Kootstra NA van der Valk M Geerlings SE Cross-sectional comparison of the prevalence of age-associated comorbidities and their risk factors between HIV-infected and uninfected individuals: the AGEhIV cohort study. Clin Infect Dis (2014) 59(12):1787–97. 10.1093/cid/ciu701 25182245

[8] Deeks SG HIV infection, inflammation, immunosenescence, and aging. Annu Rev Med (2011) 62:141–55. 10.1146/annurev-med-042909-093756 PMC3759035 21090961

[9] Deeks SG Tracy R Douek DC Systemic effects of inflammation on health during chronic HIV infection. Immunity. (2013) 39(4):633–45. 10.1016/j.immuni.2013.10.001 PMC4012895 24138880

[10] Brunner S Herndler-Brandstetter D Weinberger B Grubeck-Loebenstein B Persistent viral infections and immune aging. Ageing Res Rev (2011) 10(3):362–9. 10.1016/j.arr.2010.08.003 20727987

[11] Czesnikiewicz-Guzik M Lee WW Cui D Hiruma Y Lamar DL Yang ZZ T cell subset-specific susceptibility to aging. Clin Immunol (2008) 127(1):107–18. 10.1016/j.clim.2007.12.002 PMC2435295 18222733

[12] Fulop T Dupuis G Witkowski JM Larbi A The Role of Immunosenescence in the Development of Age-Related Diseases. Rev Invest Clin (2016) 68(2):84–91. 27103044

[13] Shaw AC Goldstein DR Montgomery RR Age-dependent dysregulation of innate immunity. Nat Rev Immunol (2013) 13(12):875–87. 10.1038/nri3547 PMC4096436 24157572

[14] Appay V Dunbar PR Callan M Klenerman P Gillespie GM Papagno L Memory CD8+ T cells vary in differentiation phenotype in different persistent virus infections. Nat Med (2002) 8(4):379–85. 10.1038/nm0402-379 11927944

[15] Appay V Fastenackels S Katlama C Ait-Mohand H Schneider L Guihot A Old age and anti-cytomegalovirus immunity are associated with altered T-cell reconstitution in HIV-1-infected patients. AIDS (2011) 25(15):1813–22. 10.1097/QAD.0b013e32834640e6 21412126

[16] Booiman T Wit FW Girigorie AF Maurer I De Francesco D Sabin CA Terminal differentiation of T cells is strongly associated with CMV infection and increased in HIV-positive individuals on ART and lifestyle matched controls. PloS One (2017) 12(8):e0183357. 10.1371/journal.pone.0183357 28806406 PMC5555623

[17] Booiman T Wit FW Maurer I De Francesco D Sabin CA Harskamp AM High Cellular Monocyte Activation in People Living With Human Immunodeficiency Virus on Combination Antiretroviral Therapy and Lifestyle-Matched Controls Is Associated With Greater Inflammation in Cerebrospinal Fluid. Open Forum Infect Dis (2017) 4(3):ofx108. 10.1093/ofid/ofx108 28680905 PMC5494939

[18] Cobos Jimenez V Wit FW Joerink M Maurer I Harskamp AM Schouten J T-Cell Activation Independently Associates With Immune Senescence in HIV-Infected Recipients of Long-term Antiretroviral Treatment. J Infect Dis (2016) 214 (2):216–25. 10.1093/infdis/jiw146 PMC8445638 27073222

[19] Hearps AC Maisa A Cheng WJ Angelovich TA Lichtfuss GF Palmer CS HIV infection induces age-related changes to monocytes and innate immune activation in young men that persist despite combination antiretroviral therapy. AIDS (2012) 26(7):843–53. 10.1097/QAD.0b013e328351f756 22313961

[20] Hunt PW Martin JN Sinclair E Bredt B Hagos E Lampiris H T cell activation is associated with lower CD4+ T cell gains in human immunodeficiency virus-infected patients with sustained viral suppression during antiretroviral therapy. J Infect Dis (2003) 187(10):1534–43. 10.1086/374786 12721933

[21] Kamat A Misra V Cassol E Ancuta P Yan Z Li C A plasma biomarker signature of immune activation in HIV patients on antiretroviral therapy. PloS One (2012) 7(2):e30881. 10.1371/journal.pone.0030881 22363505 PMC3281899

[22] Neuhaus J Jacobs DR Jr. Baker JV Calmy A Duprez D La Rosa A Markers of inflammation, coagulation, and renal function are elevated in adults with HIV infection. J Infect Dis (2010) 201(12):1788–95. 10.1086/652749 PMC2872049 20446848

[23] Westhorpe CL Maisa A Spelman T Hoy JF Dewar EM Karapanagiotidis S Associations between surface markers on blood monocytes and carotid atherosclerosis in HIV-positive individuals. Immunol Cell Biol (2014) 92(2):133–8. 10.1038/icb.2013.84 24296810

[24] Leite Pereira A Tchitchek N Lambotte O Le Grand R Cosma A Characterization of Leukocytes From HIV-ART Patients Using Combined Cytometric Profiles of 72 Cell Markers. Front Immunol (2019) 10:1777. 10.3389/fimmu.2019.01777 31447833 PMC6691046

[25] McCausland MR Juchnowski SM Zidar DA Kuritzkes DR Andrade A Sieg SF Altered Monocyte Phenotype in HIV-1 Infection Tends to Normalize with Integrase-Inhibitor-Based Antiretroviral Therapy. PloS One (2015) 10(10):e0139474. 10.1371/journal.pone.0139474 26430882 PMC4591977

[26] Karim R Mack WJ Kono N Tien PC Anastos K Lazar J T-cell activation, both pre- and post-HAART levels, correlates with carotid artery stiffness over 6.5 years among HIV-infected women in the WIHS. J Acquir Immune Defic Syndr (2014) 67(3):349–56. 10.1097/QAI.0000000000000311 PMC4197806 25314253

[27] McComsey GA Kitch D Sax PE Tierney C Jahed NC Melbourne K Associations of inflammatory markers with AIDS and non-AIDS clinical events after initiation of antiretroviral therapy: AIDS clinical trials group A5224s, a substudy of ACTG A5202. J Acquir Immune Defic Syndr (2014) 65(2):167–74. 10.1097/01.qai.0000437171.00504.41 PMC3943548 24121755

[28] Tenorio AR Zheng Y Bosch RJ Krishnan S Rodriguez B Hunt PW Soluble markers of inflammation and coagulation but not T-cell activation predict non-AIDS-defining morbid events during suppressive antiretroviral treatment. J Infect Dis (2014) 210(8):1248–59. 10.1093/infdis/jiu254 PMC4192039 24795473

[29] De Francesco D Wit FW Burkle A Oehlke S Kootstra NA Winston A Do people living with HIV experience greater age advancement than their HIV-negative counterparts? AIDS (2019) 33(2):259–68. 10.1097/QAD.0000000000002063 PMC6319574 30325781

[30] De Francesco D Wit FW Cole JH Kootstra NA Winston A Sabin CA The ‘COmorBidity in Relation to AIDS’ (COBRA) cohort: Design, methods and participant characteristics. PloS One (2018) 13(3):e0191791. 10.1371/journal.pone.0191791 29596425 PMC5875743

[31] Bürkle A Moreno-Villanueva M Bernhard J Blasco M Zondag G Hoeijmakers JH MARK-AGE biomarkers of ageing. Mech Ageing Dev (2015) 151:2–12. 10.1016/j.mad.2015.03.006 25818235

[32] Bürkle A Berthold M Junk M Moreno-Villanueva M Method for the determination of biological age in human beings. Google Patents (2016).

[33] Weber D Stuetz W Bernhard W Franz A Raith M Grune T Oxidative stress markers and micronutrients in maternal and cord blood in relation to neonatal outcome. Eur J Clin Nutr (2014) 68(2):215–22. 10.1038/ejcn.2013.263 24327121

[34] Jansen E Beekhof P Cremers J Weinberger B Fiegl S Toussaint O Quality control data of physiological and immunological biomarkers measured in serum and plasma. Mech Ageing Dev (2015) 151:54–9. 10.1016/j.mad.2015.06.004 26166476

[35] Bacalini MG Deelen J Pirazzini C De Cecco M Giuliani C Lanzarini C Systemic Age-Associated DNA Hypermethylation of ELOVL2 Gene: In Vivo and In Vitro Evidences of a Cell Replication Process. J Gerontol A Biol Sci Med Sci (2017) 72(8):1015–23. 10.1093/gerona/glw185 PMC5861890 27672102

[36] Vanhooren V Laroy W Libert C Chen C. N-glycan profiling in the study of human aging. Biogerontology (2008) 9(5):351–6. 10.1007/s10522-008-9140-z 18431686

[37] Helland I Partial least squares regression. Wiley StatsRef: Statistics Reference Online NJ, USA: John Wiley & Sons, Ltd (2006).

[38] Galindo-Prieto B Eriksson L Trygg J Variable influence on projection (VIP) for orthogonal projections to latent structures (OPLS). J Chemometrics (2014) 28(8):623–32. 10.1002/cem.2627

[39] Hearps AC Martin GE Angelovich TA Cheng WJ Maisa A Landay AL Aging is associated with chronic innate immune activation and dysregulation of monocyte phenotype and function. Aging Cell (2012) 11(5):867–75. 10.1111/j.1474-9726.2012.00851.x 22708967

[40] Seidler S Zimmermann HW Bartneck M Trautwein C Tacke F Age-dependent alterations of monocyte subsets and monocyte-related chemokine pathways in healthy adults. BMC Immunol (2010) 11:30. 10.1186/1471-2172-11-30 20565954 PMC2910032

[41] Nyugen J Agrawal S Gollapudi S Gupta S Impaired functions of peripheral blood monocyte subpopulations in aged humans. J Clin Immunol (2010) 30(6):806–13. 10.1007/s10875-010-9448-8 PMC2970801 20703784

[42] van Duin D Allore HG Mohanty S Ginter S Newman FK Belshe RB Prevaccine determination of the expression of costimulatory B7 molecules in activated monocytes predicts influenza vaccine responses in young and older adults. J Infect Dis (2007) 195(11):1590–7. 10.1086/516788 17471428

[43] van Duin D Mohanty S Thomas V Ginter S Montgomery RR Fikrig E Age-associated defect in human TLR-1/2 function. J Immunol (2007) 178(2):970–5. 10.4049/jimmunol.178.2.970 17202359

[44] Green SR Han KH Chen Y Almazan F Charo IF Miller YI The CC chemokine MCP-1 stimulates surface expression of CX3CR1 and enhances the adhesion of monocytes to fractalkine/CX3CL1 via p38 MAPK. J Immunol (2006) 176(12):7412–20. 10.4049/jimmunol.176.12.7412 16751386

[45] Funderburg NT Zidar DA Shive C Lioi A Mudd J Musselwhite LW Shared monocyte subset phenotypes in HIV-1 infection and in uninfected subjects with acute coronary syndrome. Blood (2012) 120(23):4599–608. 10.1182/blood-2012-05-433946 PMC3512236 23065151

[46] Krishnan S Wilson EM Sheikh V Rupert A Mendoza D Yang J Evidence for innate immune system activation in HIV type 1-infected elite controllers. J Infect Dis (2014) 209(6):931–9. 10.1093/infdis/jit581 PMC3935475 24185941

[47] Cheadle WG The human leukocyte antigens and their relationship to infection. Am J Surg (1993) 165(2A Suppl):75S–81S. 10.1016/S0002-9610(05)81210-3 8439003

[48] Debets JM Van de Winkel JG Ceuppens JL Dieteren IE Buurman WA Cross-linking of both Fc gamma RI and Fc gamma RII induces secretion of tumor necrosis factor by human monocytes, requiring high affinity Fc-Fc gamma R interactions. Functional activation of Fc gamma RII by treatment with proteases or neuraminidase. J Immunol (1990) 144(4):1304–10. 2137489

[49] Nolan A Kobayashi H Naveed B Kelly A Hoshino Y Hoshino S Differential role for CD80 and CD86 in the regulation of the innate immune response in murine polymicrobial sepsis. PloS One (2009) 4(8):e6600. 10.1371/journal.pone.0006600 19672303 PMC2719911

[50] Gonias SL Campana WM LDL receptor-related protein-1: a regulator of inflammation in atherosclerosis, cancer, and injury to the nervous system. Am J Pathol (2014) 184(1):18–27. 10.1016/j.ajpath.2013.08.029 24128688 PMC3873482

[51] Gorovoy M Gaultier A Campana WM Firestein GS Gonias SL Inflammatory mediators promote production of shed LRP1/CD91, which regulates cell signaling and cytokine expression by macrophages. J Leukoc Biol (2010) 88(4):769–78. 10.1189/jlb.0410220 PMC2974427 20610799

[52] Pawaria S Binder RJ CD91-dependent programming of T-helper cell responses following heat shock protein immunization. Nat Commun (2011) 2:521. 10.1038/ncomms1524 22045000 PMC3356570

[53] Zilber MT Gregory S Mallone R Deaglio S Malavasi F Charron D CD38 expressed on human monocytes: a coaccessory molecule in the superantigen-induced proliferation. Proc Natl Acad Sci U S A (2000) 97(6):2840–5. 10.1073/pnas.050583197 PMC16017 10706632

[54] Glaria E Valledor AF Roles of CD38 in the Immune Response to Infection. Cells (2020) 9(1):228. 10.3390/cells9010228 PMC7017097 31963337

[55] Kowalczyk D Macura-Biegun A Zembala M The expression of CD40 on monocytes of children with primary humoral immunodeficiencies. Pediatr Res (2006) 59(6):816–9. 10.1203/01.pdr.0000219298.96471.18 16641210

[56] Dock JN Effros RB Role of CD8 T Cell Replicative Senescence in Human Aging and in HIV-mediated Immunosenescence. Aging Dis (2011) 2(5):382–97. PMC3269814 22308228

[57] Effros RB Loss of CD28 expression on T lymphocytes: a marker of replicative senescence. Dev Comp Immunol (1997) 21(6):471–8. 10.1016/S0145-305X(97)00027-X 9463780

[58] Akbar AN Henson SM Are senescence and exhaustion intertwined or unrelated processes that compromise immunity? Nat Rev Immunol (2011) 11(4):289–95. 10.1038/nri2959 21436838

[59] Gregg R Smith CM Clark FJ Dunnion D Khan N Chakraverty R The number of human peripheral blood CD4+ CD25high regulatory T cells increases with age. Clin Exp Immunol (2005) 140(3):540–6. 10.1111/j.1365-2249.2005.02798.x PMC1809384 15932517

[60] Simone R Zicca A Saverino D The frequency of regulatory CD3+CD8+CD28- CD25+ T lymphocytes in human peripheral blood increases with age. J Leukoc Biol (2008) 84(6):1454–61. 10.1189/jlb.0907627 18780874

[61] Khaitan A Unutmaz D Revisiting immune exhaustion during HIV infection. Curr HIV/AIDS Rep (2011) 8(1):4–11. 10.1007/s11904-010-0066-0 21188556 PMC3144861

[62] van Bilsen WPH Zaaijer HL Matser A van den Hurk K Slot E Schim van der Loeff MF Infection Pressure in Men Who Have Sex With Men and Their Suitability to Donate Blood. Clin Infect Dis (2019) 68(6):1001–8. 10.1093/cid/ciy596 30052873

[63] Marchetti G Riva A Cesari M Bellistri GM Gianelli E Casabianca A HIV-infected long-term nonprogressors display a unique correlative pattern between the interleukin-7/interleukin-7 receptor circuit and T-cell homeostasis. HIV Med (2009) 10(7):422–31. 10.1111/j.1468-1293.2009.00710.x 19459995

[64] Korndewal MJ Mollema L Tcherniaeva I van der Klis F Kroes AC Oudesluys-Murphy AM Cytomegalovirus infection in the Netherlands: seroprevalence, risk factors, and implications. J Clin Virol (2015) 63:53–8. 10.1016/j.jcv.2014.11.033 25600606

[65] Kilpatrick RD Rickabaugh T Hultin LE Hultin P Hausner MA Detels R Homeostasis of the naive CD4+ T cell compartment during aging. J Immunol (2008) 180(3):1499–507. 10.4049/jimmunol.180.3.1499 PMC2940825 18209045

[66] Macallan DC Wallace D Zhang Y De Lara C Worth AT Ghattas H Rapid turnover of effector-memory CD4(+) T cells in healthy humans. J Exp Med (2004) 200(2):255–60. 10.1084/jem.20040341 PMC2212011 15249595

[67] Messi M Giacchetto I Nagata K Lanzavecchia A Natoli G Sallusto F Memory and flexibility of cytokine gene expression as separable properties of human T(H)1 and T(H)2 lymphocytes. Nat Immunol (2003) 4(1):78–86. 10.1038/ni872 12447360

